# Supplementary material for: A Global Screen for Assembly State Changes of the Mitotic Proteome by SEC-SWATH-MS
Source: Cell Syst. 2020 Feb 26;10(2):133–155.e6. doi: 10.1016/j.cels.2020.01.001 (PMC7042714; doi:10.1016/j.cels.2020.01.001)

Q9P0K7 | RAI14_HUMAN | RAI14 KIAA1334 NORPEG
Monomer MW [kDa]: 110.041 Monomer expected elution fraction: 39

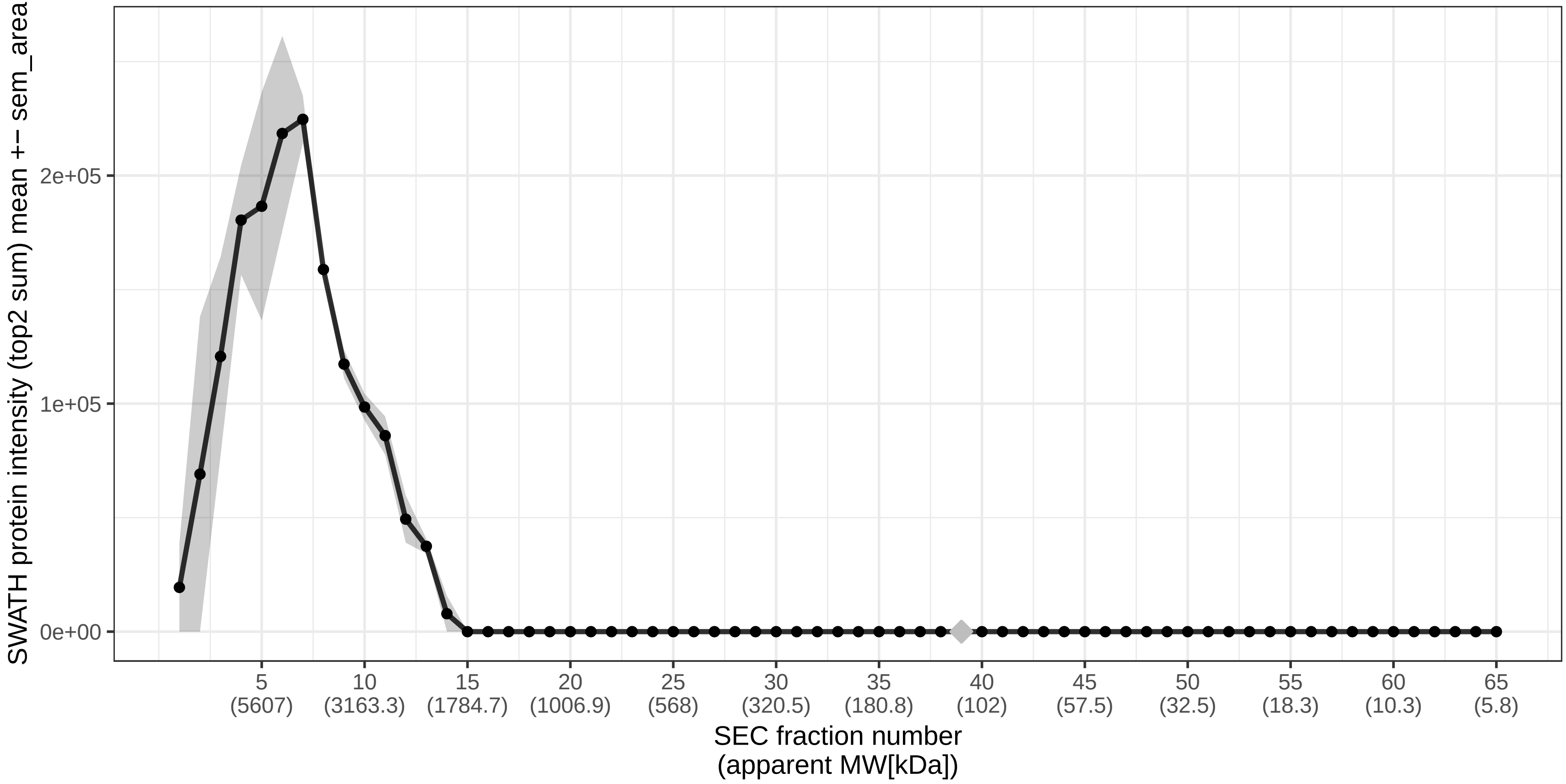

Supplement: Data S1. SEC-SWATH-MS Protein Chromatograms, Related to Figure 1 [file mmc6.zip › SECchrom_Q9P0K7_RAI14_HUMAN_RAI14_KIAA1334_NORPEG.pdf]
